# Effect of dietary chitosan supplementation on intestinal barrier function and growth performance in weaned piglets challenged by *Enterohemorrhagic Escherichia coli*

**DOI:** 10.1186/s12917-026-05815-y

**Published:** 2026-08-08

**Authors:** Jiahao Liu, Simona De Blasio, Kunhong Xie, Xiang Li, Yuheng Luo, Ping Zheng, Xiangbing Mao, Hui Yan, Quyuan Wang, Liam Good, Ludovic Pelligand, Jun He

**Affiliations:** 1 Institute of Animal Nutrition, Sichuan Agricultural University, Chengdu, Sichuan 611130 People’s Republic of China; 2 Key Laboratory of Animal Nutrition and Efficient Feed Utilization, Chengdu, Sichuan 611130 P. R. China; 3 Department of Pathobiology and Population Sciences, Royal Veterinary College, Camden, London, NW1 0TU UK; 4 Department of Comparative Biomedical Sciences, Royal Veterinary College, Hawkshead, Hatfield, AL9 7TA UK; 5 Department of Clinical Services and Sciences, Royal Veterinary College, Hawkshead, Hatfield, AL9 7TA UK

**Keywords:** *Enterohemorrhagic Escherichia coli*, Inflammation, Chitosan, Weaned pigs, Alternative to antimicrobials

## Abstract

**Background:**

*Enterohemorrhagic Escherichia coli* O157:H7 (EHEC) is an important zoonotic pathogen that disrupts intestinal epithelial barrier integrity and induces excessive inflammatory responses, leading to impaired growth performance and intestinal injury. EHEC is also an important cause hemolytic uremic syndrome (HUS) in children and older adults. In pig production, chitosan is considered a promising alternative to antibiotics due to its bioadhesive and antimicrobial properties, but the effects and underlying mechanisms of chitosan (COS) under pathogenic challenge remain to be elucidated.

**Results:**

One hundred and eight pigs were randomly divided into three treatment groups: an unchallenged control (CON), an EHEC-challenged control (ECON), and an EHEC-challenged group supplemented with 100 mg/kg COS (ECOS). Results show that EHEC challenge increased the feed conversion ratio (FCR), increased inflammatory cytokine levels, disrupted intestinal morphology, and downregulated tight junction and nutrient transporter gene expression (*P* < 0.05). Dietary COS supplementation decreased FCR during days 6–14 (*P* < 0.05) and tended to improve average daily gain (ADG) over the same period (0.05 < *P* < 0.10). Moreover, COS tended to decrease faecal shedding of total *E. coli* (*P* = 0.085) and EHEC, attenuated systemic inflammation by decreasing serum TNF-α and IL-6 levels, and enhanced humoral immunity as indicated by increased IgA and IgM concentrations (*P* < 0.05). Importantly, COS alleviated EHEC-induced intestinal injury by restoring villus height and villus-to-crypt ratio, with enhanced mucosal digestive enzyme activities, and upregulated expression of tight junction proteins (*ZO-1* and *occludin*) and nutrient transporters (*SGLT-1* and *PEPT1*) (*P* < 0.05).

**Conclusions:**

In conclusion, these findings indicate that dietary COS improves growth performance in EHEC-challenged weaned pigs, with enhanced intestinal barrier integrity and nutrient transport capacity.

**Supplementary Information:**

The online version contains supplementary material available at https://doi.org/10.1186/s12917-026-05815-y.

## Introduction

 Weaning stress represents a critical bottleneck in early post-weaning growth and development of piglets and is often accompanied by impaired intestinal barrier function and immunosuppression, thereby markedly increasing susceptibility to enteric pathogen infection. *Enterohemorrhagic Escherichia coli* O157:H7 (EHEC), an important zoonotic pathogen, can specifically adhere to the intestinal epithelial surface and secrete virulence factors, including Shiga toxins and intimin, which disrupt epithelial structural integrity. In addition, EHEC infection activates the NLRP3 signalling pathway, triggering excessive inflammatory responses characterized by elevated production of pro-inflammatory cytokines such as TNF-α and IL-6, ultimately leading to diarrhea, intestinal injury, and even mortality in piglets [1–3]. Moreover, EHEC exhibits strong environmental survival and prolonged fecal shedding, facilitating transmission within swine herds and dissemination through the food chain, thereby posing dual threats to swine production and public health [4]. Therefore, reducing intestinal colonization and shedding of EHEC during pig production is of great importance for safeguarding animal and public health.

Functional polysaccharide feed additives attract increasing attention as promising alternatives to antibiotics for improving intestinal health in animal nutrition, owing to their favorable biosafety, biodegradability, and multi-target regulatory potential [5, 6]. Chitosan, is a natural cationic polysaccharide, isolated as a byproduct from crustacea or fungi, is widely available and exhibits diverse biological activities, including antibacterial properties, adsorption of harmful substances, and improvement of intestinal function [7]. Current research has predominantly examined low molecular weight chitosan (LMWC) and chitosan oligosaccharides; however, their rapid diffusion or absorption (short-chain oligosaccharides) in the intestinal lumen may restrict their capacity to directly protect the intestinal barrier under pathogenic infection and may pose systemic exposure risks [8–10]. In contrast, higher molecular weight chitosan possesses a higher overall positive charge and pronounced bioadhesive properties, enabling the formation of a stable local functional layer on microbial and or intestinal mucosal surfaces [11–13]. Under the combined challenges of weaning stress and EHEC infection, higher molecular weight chitosan may exert intestinal protective effects by helping to limit pathogen persistence in the intestinal lumen, supporting epithelial integrity, and modulating inflammatory responses [14, 15]. Nevertheless, systematic studies evaluating the effects of higher molecular weight chitosan (250–500 kDa) on growth performance and intestinal health in weaned piglets challenged with EHEC, as well as the underlying mechanisms, remain limited. Therefore, the present study was designed to address these gaps and to clarify the application potential and mechanisms of action of COS, providing a scientific basis for its use in preventing EHEC infection and maintaining intestinal health in swine production.

## Materials and methods

The piglets were sourced from the Jiangwan Animal Husbandry Development Co., Ltd. farm, located in Xinlin Town, Ebian Yi Autonomous County, Leshan City, Sichuan Province, China. This study was conducted in strict compliance with the animal welfare regulations of the People’s Republic of China and was designed to mitigate any potential pain or distress inflicted upon experimental animals, consistent with the research’s scientific aims.

### Bacterial strain and culture

EHEC serotype O157:H7 (CICC 10907) was acquired from the China Institute of Veterinary Drug Control (Beijing, China). Luria-Bertani (LB) broth and LB agar were prepared and autoclaved at 121 °C, 0.11 MPa for 20 min (pH 6). The strain was revived in 10 mL LB broth at 37 °C with shaking for 24 h, then streaked onto LB agar. A single colony was inoculated into 50 mL LB broth, cultured overnight (37 °C and 250 rpm), followed by subculture and serial dilution on LB agar for bacterial enumeration.

### Experimental design and feed

A total of 108 piglets (Duroc🞨Landrace🞨Yorkshire) weaned at 30 days of age were randomly allocated to three treatments (18 replicates per treatment were used, and each replicate consists of 2 piglets, for each treatment the male: female ratio was 1:1): CON (pigs were fed with a basal diet), ECON (pigs were fed with a basal diet and challenged by EHEC), ECOS (pigs were fed with a basal diet containing 100 mg/kg COS and challenged by EHEC). The COS was purchased as food grade and GMP certified. It was a white to light yellow, odourless free-flowing powder, 90.34% degree of deacetylation, with a molecular weight between 250 and 500 kDa. These piglets were followed for 21 days post-weaning. On day 6 post-weaning, the challenged groups received an oral dose of 100 mL LB broth containing 1 × 10^9^ CFU/mL EHEC via orogastric gavage, while the non-challenged group was administered a same volume of sterile LB medium. The administration volume was selected based on previous studies in young piglets and our preliminary experiments to achieve a reliable and reproducible EHEC challenge model [16]. Experimental weaner diets, based on a corn-soybean meal formulation, were formulated to meet the nutrient specifications outlined by the National Research Council (NRC, 2012), contained a medium-high crude protein level without antimicrobial growth promoters or feed additives, and were prepared as mash at the SICAU research farm (ingredients and calculated composition shown in supplementary Table 1. COS is homogenized via a two-step process: dilution with extruded corn to form a premix, followed by mixing with other ingredients.

### Animal housing and management

Piglets were housed two per 3.6 m² in slatted pens equipped with rubber insulation boards, a three-place feeder, and two nipple drinkers, in an environmentally controlled room with mechanical ventilation; temperature (28 °C in weeks 1–2, then reduced by 1.5 °C per week to 25 °C) and relative humidity (60 ± 5%) were thermostatically controlled and monitored daily, aerial ammonia was measured monthly, and empty pens and plastic boards were used to prevent cross-contamination between groups.

### Body weight, feed intake, and feed conversion ratio

Individual pig body weight was recorded at weaning, day 7, 14, and 21 post-weaning. Average daily gain (ADG, g/pig/d) and feed conversion ratio (FCR) were calculated on a pen level. Feed allowance and refusals were recorded per pen. Average Daily feed intake (ADFI, g/pig/d) was calculated for each experimental phase.

### Faecal sampling

Faeces were sampled on pen level at day 0, 7, 14, and 21 post-weaning (challenged on day 6). The sample was put in sterile cups and kept on ice until the lab. The pooled sample was homogenized with a sterile tool and 2 × 1 gram of faeces were stored in 2 mL cryotubes at -20 °C until analysis (e.g. *E. coli* shedding). For cultivation, only faecal samples collected at d0, d7, d14, and d21 were utilized. Measurements of *E. coli* shedding (on pen level, *n* = 9) were performed meeting to the method as described by Busser [17], the method consists in the isolation of haemolytic *E. coli* and enumeration of total *E. coli* (log colony forming units (CFU) per mL); the specific media required for the cultivation of *E. coli* and EHEC were procured from Haber Biotechnology Ltd (Qingdao, China). Specifically, total *E. coli* were cultured on MacConkey agar (Cat no. HB6238), while EHEC were cultured on a modified sorbitol MacConkey agar (Cat no. HB0121-3) supplemented with modified sorbitol MacConkey agar additive (Cat no. HB0121-2). Because faecal bacterial culture, serial dilution, and colony enumeration are labor-intensive and time-consuming procedures, nine pens were randomly selected from each treatment before the experiment and consistently sampled throughout the study for faecal bacterial enumeration. Fresh faeces from both piglets within each selected pen were collected and thoroughly pooled to obtain one representative sample for bacterial culture and enumeration.

### Blood sampling

At the experiment’s end, blood specimens were harvested after 12 h fasting by jugular vein puncture (in the morning on d 21 post-weaning). Whole blood was transferred into two 10 mL vacuum collection tubes (approximately 7.5 mL of whole blood per tube); serum was subsequently isolated by centrifugation at 3,500×g for 15 min, and the resulting serum aliquots were stored at -20 °C until subsequent biochemical analysis. Enzyme-linked immunosorbent assay (ELISA) kits were used for the quantification of serum inflammatory cytokines and immune globulins: Porcine TNF-α ELISA Kit (MM-0422O1), Porcine IL-1β ELISA Kit (MM-0863O1), Porcine IL-6 ELISA Kit (MM- MM-0864O1), and Porcine IgA, IgG, and IgM ELISA Kits (catalog nos. MM-0905O1, MM-0403O1, MM-0402O1) were all purchased from Jiangsu Enzyme-linked Biotechnology Co., Ltd. (Jiangsu, China). The indicators were measured according to the manufacturer’s instructions. Others serum samples were transported to Ya’an People’s Hospital (Ya’an, China) for hematological parameter assessment (e.g. total protein, albumin, globulin, glucose).

### Tissue sampling

Intestinal tissue collection was conducted subsequent to blood sampling. Piglets were euthanized by intravenous injection of sodium pentobarbital at 200 mg/kg BW, and then each segment of the small intestine were dissected-out. The middle segments of the intestinal tissues 4 cm obtained were gently flushed with ice-cold PBS, followed by fixation in 4% paraformaldehyde solution for morphological analyses. The intestinal mucosa was obtained from the residual intestinal segments with scalpel blade and place in frozen tube then frozen by immersion in liquid nitrogen and stored at -80 °C until analysis (e.g. lactase, amylase, sucrase, alkaline phosphatase, endotoxin, and nutrient absorption and transportation related expression gene expression levels).

#### Intestinal morphology and histological analysis

Fixed small intestinal segments were dewaxed with graded anhydrous ethanol, stained by hematoxylin and eosin (H&E), and sealed with neutral resin as described previously [18]. The sections were then analyzed for crypt depth (CD), villus height (VH), and VH/CD ratio using an Image-Pro Plus 6.0 image analysis system (Image-Pro Plus 6.0, Media Cybernetics, Maryland, USA. Pathological scores of intestinal tissues were determined by the Chiu scoring system on H&E-stained small intestinal sections [19]., detailed criteria are listed in Supplementary data.

#### Enzyme activity and endotoxin

The intestinal mucosa was homogenized with saline, and then the supernatants were isolated and utilized to determine the enzyme activities such as the lactase (Porcine lactase ELISA Kit MM-MM-1746O1), invertase (Porcine invertase ELISA Kit MM-1747O1), and maltase (Porcine maltase ELISA Kit MM-1748O1), Alkaline phosphatase activity (Porcine ALP ELISA Kit MM-0634O1) and endotoxin content (Porcine ET ELISA Kit MM- MM-0635O1) were also determined by using specific enzyme-linked immunosorbent assay kits (Jiangsu Meimian industrial Co., Ltd.). All procedures were performed according to the instructions.

#### qPCR quantification

Total mucosal RNA (18 samples per group were processed for qPCR analysis) was extracted with TRIzol and reverse-transcribed to cDNA using a PrimeScript™ RT kit with gDNA Eraser (Takara), under conditions of 37 °C for 15 min and 85 °C for 5 s. qPCR was performed with SYBR^®^ Green reagents (Takara) using the program: 95 °C for 30 s, 40 cycles of 95 °C for 5 s and 60 °C for 30 s. Target gene expression was analyzed by the 2^−ΔΔCt^ method with β-actin as the internal control [20], and all primers are listed in supplementary Table 2. Representative qPCR melt curves are presented in Supplementary Figure S1.

### Statistical analysis

The experimental data was analysed using one-way analysis of variance (ANOVA) via the General Linear Model function of SPSS 24.0 (SPSS, Inc). Significant differences were declared at *P* < 0.05, with near significant trends as 0.05 < *P* ≤ 0.10. Significant fixed effects were further analysed by Least Significant Differences (LSD, Fisher’s LSD method) to compare treatment means. Data is presented as Mean ± SEM. The *P*-value, LSD, and standard error of the mean (SEM) are reported per response parameter.

### Unexpected issues

There were no unexpected issues excepting the African Swine Fever. However, we have collected saliva samples from pigs and sent it for virus detection (samples were detected by authoritative testing organization), and only non-infected pigs were allowed to enter the research farm. Moreover, stringent disinfection procedures and management were performed throughout the trial.

## Results

### Animal health and mortality

A total of 108 piglets were included in this experiment at the weaning stage and were followed up for 3 weeks. During the experimental period, all pigs were maintained in normal feeding, drinking and mental status without any medical intervention, and no deaths occurred (zero mortality).

### Growth performance

As shown in Table 1, the FCR was significantly higher in the ECON group than in the CON and ECOS group (*P* < 0.05), whereas the ADG in the ECOS group tended to be higher than that in the ECON group (0.05 < *P* < 0.10). However, there were no differences in ADFI and BW amongst the three group during the experiment period.

**Table 1:** Effect of COS on growth performance in weaned pigs upon EHEC challenge

Items	Treatment			SEM^1^	LSD^2^	*P* - value
	CON	ECON	ECOS			
Body weight, kg						
Day 0	9.28	9.34	9.16	0.05	0.10	0.273
Day 6	10.35	10.37	10.27	0.06	0.12	0.790
Day 14	11.96	11.91	12.20	0.11	0.22	0.565
Day 21	14.42	14.49	15.10	0.19	0.38	0.261
ADG, g/d/piglet						
Day 0–6	178.47	170.83	185.74	5.50	10.73	0.558
Day 6–14	202.35	193.47	240.27	9.37	18.50	0.087
Day 14–21	350.00	368.18	414.84	17.03	34.10	0.267
ADFI, g/d/piglet						
Day 0–6	344.86	350.00	338.14	15.21	30.51	0.842
Day 6–14	412.27	441.67	452.48	15.21	30.51	0.542
Day 14–21	383.38	402.38	403.48	11.38	22.83	0.730
FCR						
Day 0–6	1.97	2.05	1.86	0.04	0.08	0.166
Day 6–14	2.08^b^	2.30^a^	1.90^b^	0.05	0.10	0.001
Day 14–21	1.70	1.72	1.60	0.05	0.10	0.567

Different superscripts (ab) within a row indicate a significant difference between treatments (*P* < 0.05). ^1^ SEM is standard error of means, *n* = 18. ^2^ LSD is least significant difference at α < 0.05 (Fisher’s LSD method). The following tables and figures are the same. *ADFI* Average daily feed intake, *ADG* Average daily gain, *FCR* Feed conversion ratio

### *E. coli* and haemolytic *E. coli* shedding

**Fig. 1:**
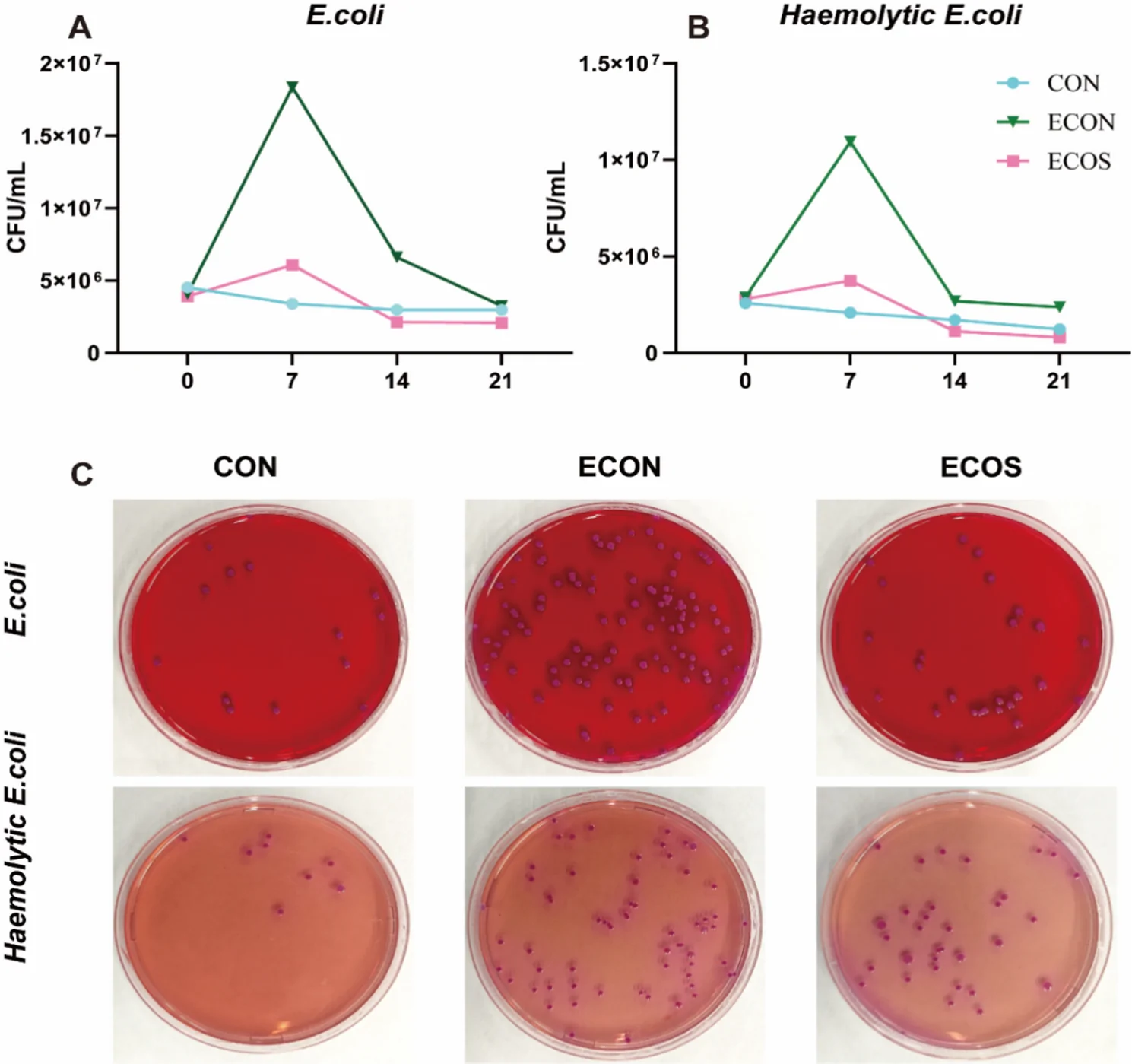
Effects of COS on CFU counts of rectal total *E. coli* and *haemolytic E. coli* shedding in weaned pigs after EHEC challenge. **A** and (**B**) show the growth of these two *E. coli* populations from 0 to 21 days respectively; (**C**) illustrates the growth of the two populations on selective media at day 7. The CON plate represents the 10⁴ fold dilution, whereas the ECON and ECOS plates represent the 10⁵ fold dilution

Pigs in the ECON and ECOS groups were challenged by EHEC at day 6 (the CON group was infused by LB culture medium). As shown in Fig. 1, there were no differences in the shedding of total *E coli* and EHEC amongst the three groups at day 0. Maximal shedding in the ECON and ECOS groups was observed at day 7, and the shedding of total *E coli* and EHEC continuously decreased from day 14 to day 21. As compared to the ECON group, dietary COS supplementation decreased shedding of the total *E coli* and EHEC in the ECOS group at day 7 (*P* = 0.085) and day 14 (*P* > 0.05) (Fig. 1). However, there were no significant differences amongst the three group (This may attribute to the big variations during the cultivation).

### Clinical serum biochemical parameters

As shown in Table 2, there were no differences in the serum concentrations of serum albumin, globulin, and glucose amongst the three groups. However, EHEC challenge decreased the serum concentration of TBILI, and decreased the serum activities of AST and CK (*P* < 0.05).

**Table 2:** Effect of COS on clinical biochemical parameters upon EHEC challenge

Items	Treatment			SEM^1^	LSD^2^	*P* - value
	CON	ECON	ECOS			
Total protein(g/L)	47.06	48.39	47.78	0.50	1.00	0.563
Albumin(g/L)	18.28	18.56	18.56	0.34	0.68	0.929
Globulin(g/L)	28.78	29.83	28.67	0.52	1.04	0.611
Glucose(mmol/L)	8.35	8.09	8.24	0.28	0.56	0.932
BUN(mmol/L)	3.56	3.52	3.50	0.11	0.22	0.974
TC(mmol/L)	2.27	2.90	2.26	0.19	0.38	0.285
Creatinine(µmol/L)	91.67	91.17	89.00	1.48	2.97	0.744
TBILI(µmol/L)	0.97^a^	0.64^b^	0.64^b^	0.05	0.10	0.005
CRP(mg/L)	5.77	5.64	4.96	0.44	0.88	0.728
ALT(U/L)	52.67	50.61	46.61	1.59	3.19	0.289
AST(U/L)	55.83^a^	43.39^b^	44.56^b^	2.17	4.35	0.037
GGT(U/L)	43.39	34.61	30.06	2.14	4.29	0.113
ALP(IU/L)	206.72	201.94	221.22	7.74	15.53	0.580
LDH(U/L)	572.50	527.78	468.94	26.30	52.76	0.277
CK(U/L)	727.75^a^	484.94^b^	539.69^b^	34.22	68.65	0.009

*ALP* Alkaline phosphatase, *BUN* Blood urea nitrogen, *ALT* Alanine aminotransferase, *AST* Aspartate aminotransferase, *TC* Total cholesterol, *GGT* Gamma-Glutamyl Transferase, *LDH* Lactate Dehydrogenase, *CK* Creatine Kinase, *CRP* C-Reactive Protein. *N* = 18, the following tables and figures are the same

### Serum inflammatory cytokines, immunoglobulins, and endotoxins

**Fig. 2:**
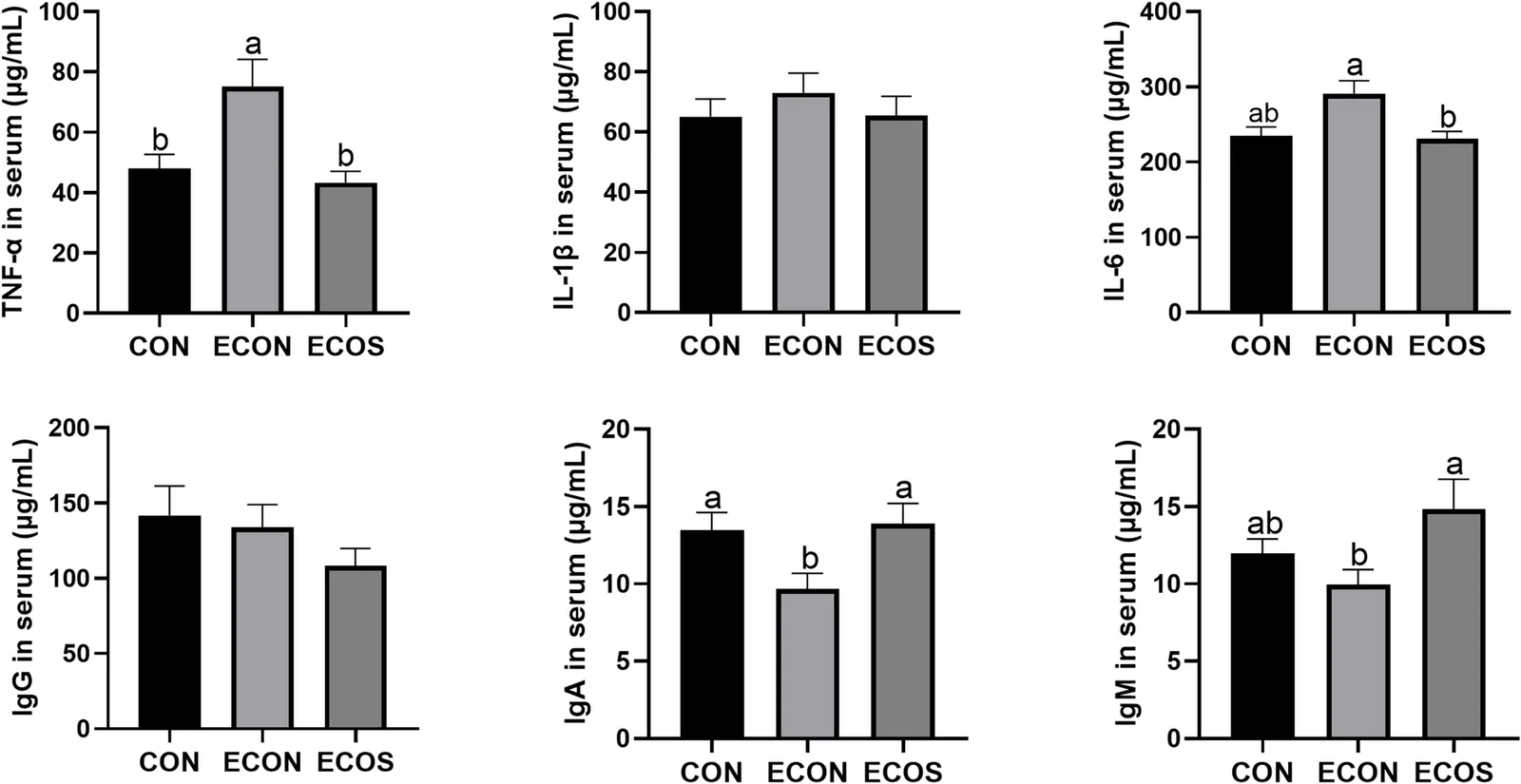
Effect of COS on serum inflammatory cytokines, immunoglobulins, and endotoxin in weaned pigs upon EHEC challenge. IgA: Immunoglobulin A, IgG: Immunoglobulin G, IgM: immunoglobulin M, IL-1β: interleukin1-β, IL-6: interleukin-6, TNF-α: tumor necrosis factor-α

As shown in Fig. 2, EHEC challenge significantly increased the serum concentration of TNF-α. However, dietary COS supplementation not only decreased the serum concentrations of TNF-α and IL-6, but also increased concentrations of immunoglobulins such as the IgA and IgM in the EHEC-challenged pigs (*P* < 0.05).

### Intestinal morphology and pathological scores

**Fig. 3:**
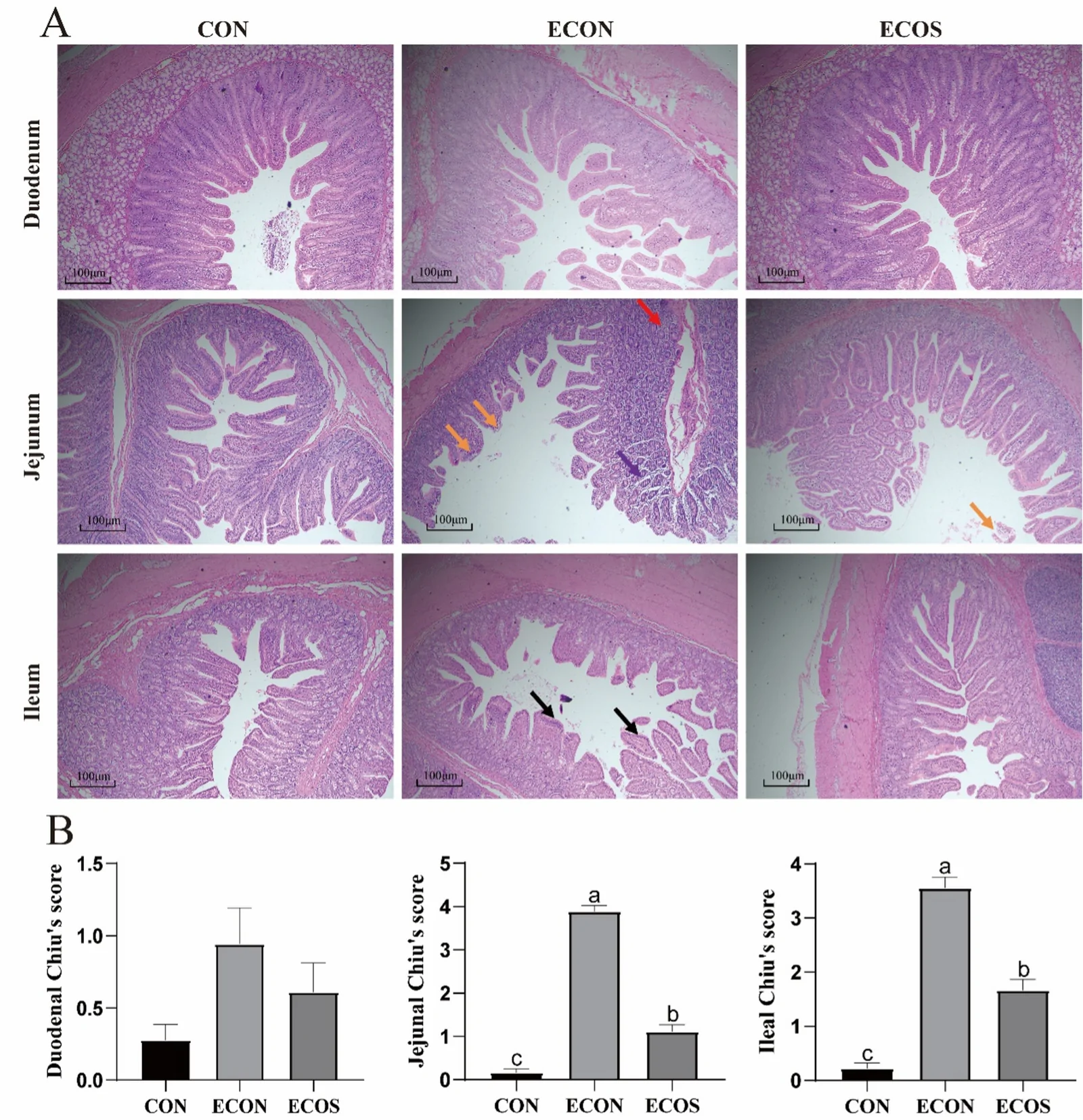
Intestinal morphology and pathological scores in weaned pigs challenged with EHEC (H&E; × 40, represent pictures for the villus). **A** Representative H&E-stained sections of duodenum, jejunum, and ileum. However, in the ECON group, the intestinal lesions were obvious and some intestinal villi were necrotic and shed, or even disappeared, especially in the jejunum. In the duodenum, jejunum and ileum in the ECOS group there were no obvious intestinal lesions and only a few of the intestinal villi were necrotic and shed. Orange arrows indicate epithelial cell shedding; purple arrows indicate edema; red arrows indicate inflammatory cell infiltration; and black arrows indicate epithelial cell necrosis. **B** Intestinal pathological scores based on Chiu’s scoring system

As shown in Table 3 and Fig. 3, EHEC challenge decreased the V/C in small intestine (duodenum, jejunum, and ileum). However, dietary COS supplementation significantly increased villus height and the V/C in the ECOS group (*P* < 0.05). Chiu’s scores were increased after EHEC challenge and were reduced by dietary COS supplementation, particularly in the jejunum and ileum (*P* < 0.05).

**Table 3:** Effect of COS on intestinal morphology in weaned pigs upon EHEC challenge

Items	Treatment			SEM^1^	LSD^2^	*P* - value
	CON	ECON	ECOS			
Duodenum						
Villus height (µm)	553.97^a^	513.41^b^	597.64^a^	10.01	20.08	0.001
Crypt depth (µm)	365.92^ab^	413.85^a^	338.00^b^	9.26	18.58	0.021
V/C	1.64^a^	1.33^b^	1.84^a^	0.04	0.08	0.001
Jejunum						
Villus height (µm)	491.34^a^	434.46^b^	517.64^a^	9.24	18.54	0.001
Crypt depth (µm)	239.62^b^	323.21^a^	252.75^b^	7.42	14.88	0.001
V/C	2.19^a^	1.40^b^	2.19^a^	0.06	0.12	0.001
Ileum						
Villus height (µm)	482.52^a^	350.00^b^	440.55^a^	10.87	21.81	0.001
Crypt depth (µm)	207.84^b^	226.86^a^	192.36^b^	3.96	7.94	0.001
V/C	2.33^a^	1.62^b^	2.35^a^	0.07	0.14	0.001

V/C: the ratio of villus height to crypt depth

### Expression of epithelial structure-related genes

**Fig. 4:**
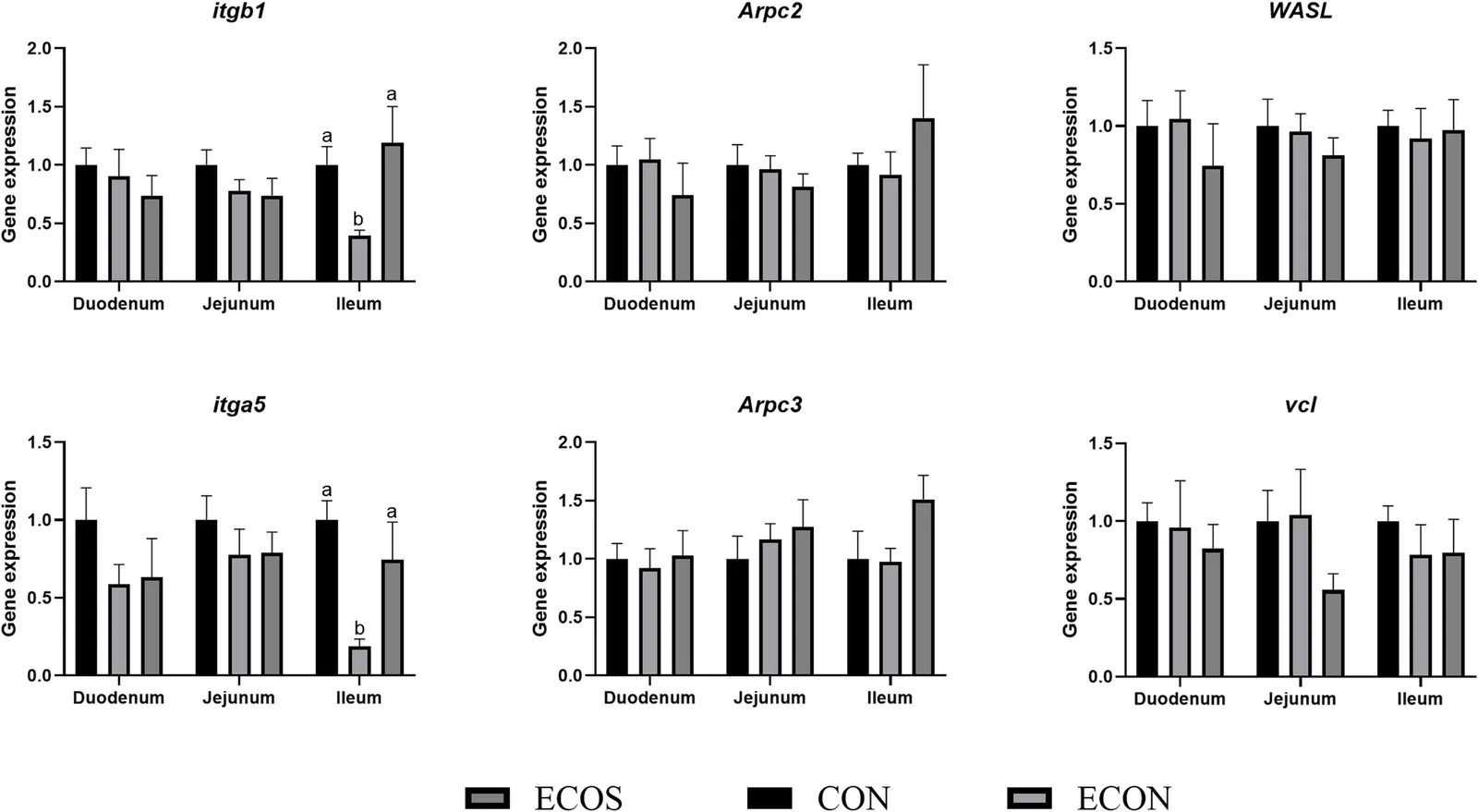
Effects of COS on the expression of epithelial structure-related genes; *itga5*: integrin alpha-5; *itgb1*: integrin beta-1; *vcl*: vinculin; WASL: wiskott–aldrich syndrome protein-like ; *Arpc2*: actin-related protein 2/3 complex subunit 2; *Arpc3*: actin-related protein 2/3 complex subunit 3

As shown in Fig. 4, EHEC challenge significantly downregulated the expression levels of *itga5* and *itgb1* in the ileal mucosa (*P* < 0.05). Dietary COS supplementation alleviated these changes by significantly increasing *itga5* and *itgb1* expression in ECOS group (*P* < 0.05).

### Intestinal mucosal enzyme protein concentration

As shown in Table 4, EHEC challenge resulted in decreases in the concentration of alkaline phosphatase (ALP), maltase, invertase, and lactase in the jejunal mucosa. However, dietary COS supplementation significantly increased their concentration in the ECOS group (*P* < 0.05).

**Table 4:** Effect of COS on intestinal mucosal enzyme protein concentration upon EHEC challenge

Items	Treatment			SEM^1^	LSD^2^	*P* - value
	CON	ECON	ECOS			
Duodenum						
ALP (ng/L)	67.58	77.94	71.25	2.78	5.58	0.310
Maltase (ng/L)	495.79	433.33	429.91	26.90	53.96	0.540
Invertase (ng/L)	212.10	212.05	259.84	13.11	26.30	0.229
Lactase (pg/ml)	179.69	188.05	193.51	9.92	19.90	0.854
ET (ng/L)	109.08	116.21	113.23	5.41	10.85	0.868
Jejunum						
ALP (ng/L)	102.33^a^	64.28^b^	77.27^b^	3.79	5.58	0.001
Maltase (ng/L)	366.09^a^	289.59^b^	353.28^a^	12.77	53.96	0.029
Invertase (ng/L)	256.62^a^	176.66^b^	226.82^a^	8.43	31.69	0.001
Lactase (pg/ml)	196.92^a^	136.73^b^	174.23^a^	7.64	19.90	0.003
ET (ng/L)	127.68	108.66	109.61	4.61	10.85	0.297
Ileum						
ALP (ng/L)	32.48	29.98	32.75	2.08	4.17	0.840
Maltase (ng/L)	286.48	310.67	286.73	11.76	23.59	0.637
Invertase (ng/L)	147.02	150.37	162.61	3.98	7.98	0.246
Lactase (pg/ml)	197.01	210.03	230.25	3.98	7.98	0.298
ET (ng/L)	101.03	105.04	117.68	2.80	5.62	0.128

*ALP* A Lkaline Phosphatase, *ET* Endotoxin

### Expressions of critical functional genes

**Fig. 5:**
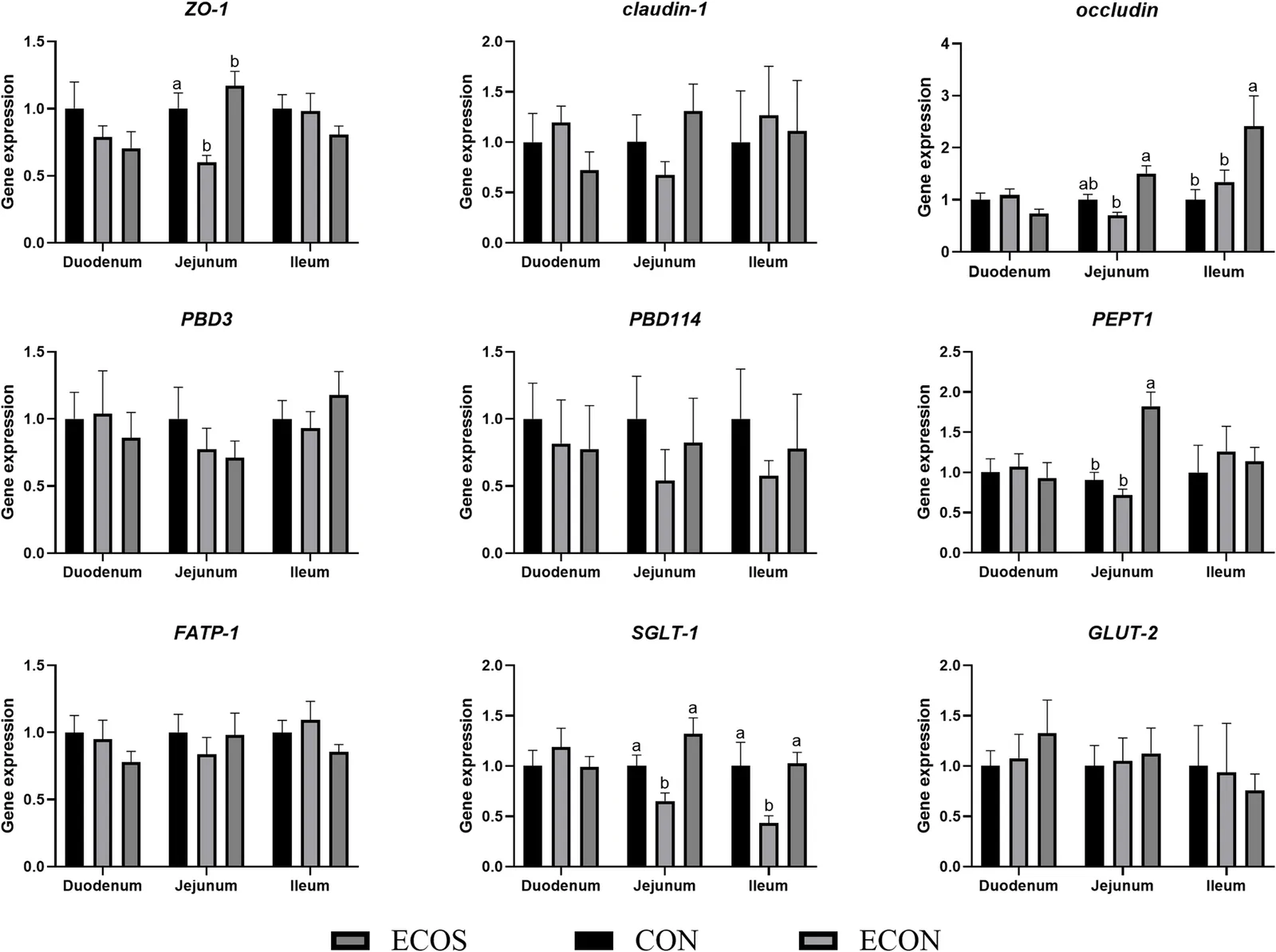
Effects of COS on the expression of intestinal epithelial barrier-related genes; *GLUT-2*: Glucose Transporter-2; *SGLT-1*: Sodium-Glucose Linked Transporter-1; *FATP-1*: Fatty Acid Transport Protein-1; *ZO-1*: Zonula Occludens-1

As shown in Fig. 5, EHEC challenge downregulated the nutrient transporters and intestinal barrier related genes expression levels such as the *SGLT-1*, *ZO-1* and *Occludin* in the jejunal mucosa (*P* < 0.05). However, dietary COS supplementation significantly upregulated their expression levels in the ECOS group (*P* < 0.05). Dietary COS supplementation elevated the jejunal *PEPT1* and ileal *SGLT-1* gene expression levels (*P* < 0.05).

## Discussion

EHEC challenge impairs intestinal barrier function and growth performance in weaned piglets, whereas the bioadhesive and cationic properties of higher molecular weight chitosan suggest its potential to protect intestinal homeostasis under pathogen challenge [21]. In this study, dietary COS (250–500 kDa) supplementation markedly alleviated the adverse effects of EHEC challenge on growth performance in weaned piglets. Specifically, during the post-challenge period from days 6 to 14, dietary COS supplementation tended to improve ADG and significantly lower FCR compared with the challenged group. The improvement in growth performance was accompanied by a sustained reduction in fecal shedding of total *E. coli* and hemolytic *E. coli.* Although the differences between groups did not reach statistical significance at all sampling points, a consistent and clear trend toward reduced colonization was observed in the COS -treated group on days 7 and 14 post-infection. These findings suggest that COS may reduce the detrimental effects of EHEC by interfering with pathogen colonization and persistence within the intestinal lumen, which is consistent with a previous study showing that dietary chitosan microparticles reduced the shedding of E. coli O157:H7 in cattle [22]. The inhibitory effects of COS on EHEC colonization are unlikely to result from a single antimicrobial mechanism but may instead involve multiple complementary modes of action. First, amino groups on the chitosan molecule chelate metal ions such as Ca²⁺ and Mg²⁺, which are essential for bacterial outer barrier integrity, cell growth and virulence expression, thereby altering the luminal microenvironment and disrupting bacterial metabolic activity and toxin production [23]. Second, as a positively charged polymer, COS may interact with negatively charged bacterial surfaces, which could influence microbial behavior within the intestinal lumen and potentially contribute to reduced intestinal epithelial disturbance under EHEC challenge. In addition, these interactions may compromise membrane integrity and permeability, leading to leakage of intracellular contents and impaired bacterial viability [24]. Importantly, by helping to maintain intestinal epithelial homeostasis under EHEC challenge, COS may attenuate mucosal inflammation and tissue disruption, thereby reducing the energetic and nutritional costs associated with immune activation and tissue repair. Consequently, a greater proportion of nutrients can be allocated to growth-related physiological processes, ultimately resulting in improved growth performance.

Immunoglobulins are key antibacterial effector molecules of the host immune system that mediate pathogen clearance and reduce pathogenicity by forming antigen–antibody complexes and subsequently neutralizing microbial toxins, inhibiting pathogen adhesion and colonization, activating the complement system, and enhancing neutrophil- and macrophage-mediated phagocytosis [25, 26]. In the present study, COS supplementation significantly attenuated the EHEC-induced decreases in serum IgM, and IgA levels, indicating an enhanced anti-infective capacity in weaned piglets. Moreover, pathogen invasion activates inflammatory signaling pathways, leading to excessive release of pro-inflammatory cytokines to initiate immune responses, whereas sustained or excessive inflammation may exacerbate tissue damage. Our results show that EHEC infection markedly increased serum TNF-α and IL-6 levels in piglets, while dietary COS supplementation effectively suppressed the elevation of these pro-inflammatory cytokines, further suggesting a potential role of COS in maintaining immune homeostasis and alleviating inflammatory responses. These are consistent with previous studies demonstrating that enterotoxigenic Escherichia coli exacerbates inflammation via the TLR4/NF-κB signaling pathway, whereas chitosan mitigates inflammatory responses by suppressing TLR4 expression and subsequently blocking downstream inflammatory signaling cascades [27, 28].

The intestine is a key organ for nutrient digestion and absorption, and the integrity of its epithelial structure and function is needed for efficient nutrient utilization and provide a first line of defence against the invasion of harmful microorganisms [16, 29]. Previous studies have shown that ETEC can colonize the small intestine and secrete exotoxins, thereby disrupting intestinal morphology [30]. In contrast, EHEC primarily delivers effector proteins, including Tir and EspA/B/D, into host epithelial cells via the type III secretion system encoded by the locus of enterocyte effacement (LEE). These effectors mediate Intimin-Tir-dependent intimate adherence and induce actin cytoskeleton rearrangement, leading to the formation of characteristic attaching and effacing (A/E) lesions, which compromise epithelial structural integrity and exacerbate local inflammatory responses [31, 32]. In this study, EHEC challenge reduced villus height and the V/C ratio while increasing crypt depth in the small intestine of piglets, indicating a pronounced impairment of the intestinal absorptive surface area and functional status. Notably, EHEC challenge also markedly downregulated the expression of *ITGA5* and *ITGB1* in the intestinal epithelium. Integrin α5β1, encoded by *ITGA5* and *ITGB1*, is a key mediator of epithelial cell-basement membrane adhesion and plays an essential role in maintaining epithelial structural stability and villus architecture [33, 34]. The decreased expression of these integrin subunits suggests that EHEC infection weakens epithelial anchorage to the basement membrane. This molecular alteration is consistent with the pathological features of A/E lesions, which involve epithelial cytoskeletal rearrangement and disruption of adhesion structures, and may, at least in part, exacerbate villus shortening and the loss of intestinal absorptive surface area. However, dietary supplementation with COS markedly alleviated these morphological alterations, as evidenced by increased villus height and V/C ratio, accompanied by a concurrent restoration of *ITGA5* and *ITGB1* expression. These findings suggest that COS may help preserve epithelial structural stability during EHEC challenge, potentially in association with improved epithelial-basement membrane integrity. Alterations in intestinal morphology are often accompanied by changes in brush-border digestive enzyme activities. Consistent with this notion, EHEC infection significantly reduced the activities of alkaline phosphatase, maltase, sucrase, and lactase, whereas COS supplementation effectively mitigated these reductions, further supporting the role of COS in preserving overall intestinal digestive and absorptive homeostasis.

Intestinal barrier integrity and nutrient transport capacity are critical determinants of growth performance in weaned piglets under enteric pathogen challenge [35, 36]. The present results demonstrate that dietary supplementation with COS markedly preserved the expression of the tight junction proteins ZO-1 and occludin in EHEC-challenged piglets, indicating effective protection of epithelial structural integrity and a reduced risk of increased paracellular permeability and trans-epithelial passage of pathogen-associated factors. Maintenance of tight junction architecture is essential for limiting pathogen translocation and preventing excessive immune activation, processes that are widely recognized as major contributors to growth impairment during the post-weaning period [32, 37]. Moreover, COS supplementation increased the gene expression levels of the nutrient transporters *SGLT-1* and *PEPT1* in both the jejunum and colon. *SGLT-1* is the key transporter mediating the active absorption of glucose and galactose across the brush-border membrane of the small intestine, whereas PEPT1 is primarily responsible for the uptake of dipeptides and tripeptides and represents the major pathway for the absorption of protein digestion products in the intestine [38, 39]. Collectively, these findings suggest that the beneficial effects of COS are not solely attributable to antimicrobial activity, but may also involve support of intestinal luminal homeostasis and epithelial integrity under EHEC challenge. Owing to its cationic and bioadhesive properties, higher molecular weight chitosan may contribute to maintaining a more favorable intestinal environment and limiting pathogen persistence within the intestinal lumen. Such effects may help reduce epithelial disturbance, preserve tight junction integrity, and support nutrient transporter expression and absorptive function. However, the present study does not provide direct evidence for reduced bacterial adhesion or physical coating of the mucosal surface, and these possibilities warrant further investigation. Future studies should investigate different supplementation levels and molecular weight ranges of chitosan and incorporate gut microbiota sequencing to further elucidate its protective effects against EHEC infection.

The EHEC challenge significantly reduced serum TBILI, AST, and CK, yet COS supplementation failed to restore these parameters. EHEC infection is generally initiated after oral exposure and intestinal colonization, with systemic complications, when they occur, arising from bacterial virulence factors and host inflammatory responses rather than from primary hepatotropic or myotropic tropism [1, 40]. TBILI reflects bilirubin metabolism and hepatobiliary function, whereas AST and CK are widely recognized as indicators of cellular integrity and tissue metabolism in multiple organs, including the liver, skeletal muscle, heart, and other metabolically active tissues. Therefore, the concurrent reductions in these biomarkers, together with the absence of abnormalities in other liver- and kidney-related biochemical indices, suggest that the present EHEC challenge induced mild systemic metabolic alterations without causing overt organ injury. The inability of COS to normalize these parameters is consistent with its high molecular weight (250–500 kDa) and bioadhesive properties, which confine its primary activity to the intestinal lumen and mucosal surface, thereby limiting systemic absorption and direct modulation of circulating biochemical markers [11, 12]. These findings further support that the protective effects of COS are predominantly localized to the gastrointestinal tract. Nevertheless, the biological significance of the reductions in TBILI, AST, and CK remains unclear. Future studies incorporating comprehensive analyses of bacterial virulence characteristics, tissue pathology, and systemic metabolic responses are warranted to clarify the mechanisms underlying these biochemical alterations and to better define the systemic reach of dietary chitosan interventions.

## Conclusion

In summary, dietary COS supplementation (100 mg/kg) improved growth performance and tended to decrease the shedding of total *E coli* and *haemolytic E. coli* in the weaned pigs following EHEC challenge. Moreover, COS inclusion in feed alleviated inflammatory responses and disruption of the intestinal epithelium in the EHEC-challenged pigs.

## Supplementary Information

Supplementary Material 1.

## Data Availability

Data can be made available upon reasonable request to the authors.

## Abbreviations

ADFI: Average daily feed intake
ADG: Average daily gain
FCR: Feed conversion ratio
ALP: Alkaline phosphatase
BUN: Blood urea nitrogen
ALT: Alanine aminotransferase
AST: Aspartate aminotransferase
TC: Total cholesterol
GGT: Gamma-Glutamyl Transferase
LDH: Lactate Dehydrogenase
CK: Creatine Kinase
CRP: C-Reactive Protein
IgA: Immunoglobulin A
IgG: Immunoglobulin G
IgM: Immunoglobulin M
IL-1β: Interleukin1-β
IL-6: Interleukin-6
qPCR: Quantitative real-time polymerase chain reaction
TNF-α: Tumor necrosis factor-α
*itga5*: Integrin alpha-5
*itgb1*: Integrin beta-1
*vcl*: Vinculin
*WASL*: Wiskott–aldrich syndrome protein-like (n-wasp)
*Arpc2*: Actin-related protein 2/3 complex subunit 2
*Arpc3*: Actin-related protein 2/3 complex subunit 3
*GLUT-2*: Glucose Transporter-2
*SGLT-1*: Sodium-Glucose Linked Transporter-1
*FATP-1*: Fatty Acid Transport Protein-1
*ZO-1*: Zonula Occludens-1
