# Supplementary material for: Effect of dietary chitosan supplementation on intestinal barrier function and growth performance in weaned piglets challenged by Enterohemorrhagic Escherichia coli
Source: BMC Vet Res. 2026 Aug 8;22:565. doi: 10.1186/s12917-026-05815-y (PMC13628782; doi:10.1186/s12917-026-05815-y)
Supplement: Supplementary file 1 [file 12917_2026_5815_MOESM1_ESM.docx]

**Histological scoring systems of intestinal tissues**

Two pathologists evaluated the degree of intestinal mucosal injury independently, the damage was graded as following: normal intestinal mucosal villi (0 points); development of subepithelial Gruenhagen’s space, usually seen at the apex of the villus, often with capillary congestion (1 points); extension of the subepithelial Gruenhagen’s space with moderate lifting of epithelial layer from the lamina propria (2 points); massive epithelial lifting down the sides of villi, with a few tips being denuded (3 points); denuded villi with lamina propria and dilated capillaries exposed, increased cellularity of lamina propria (4 points); and digestion and disintegration of lamina propria, hemorrhage, and ulceration (5 points).

Supplementary Table 1 Composition and nutrient level of the experimental diets (%)

| **Ingredients** | **Content（%）** | **Nutrient level** | **Contents** |
| --- | --- | --- | --- |
| Corn | 29.23 | DE, Mcal/kg | 3.54 |
| Extruded corn | 30.32 | CP, % | 18.46 |
| Soybean meal | 8.01 | Ca, % | 0.75 |
| Extruded soybean | 7.06 | Total phosphorus, % | 0.56 |
| Fish meal | 4.00 | AP, % | 0.37 |
| Whey powder | 5.00 | SID Lys, % | 1.31 |
| Broken rice | 3.82 | SID Met, % | 0.38 |
| Soybean protein concentrate | 6.40 | SID Thr, % | 0.76 |
| Soybean oil | 1.92 | SID Trp, % | 0.21 |
| Sucrose | 2.00 |  |  |
| Limestone | 0.62 |  |  |
| Dicalcium phosphate | 0.46 |  |  |
| NaCl | 0.20 |  |  |
| L-Lys.HCl | 0.36 |  |  |
| DL-Met | 0.07 |  |  |
| L-Thr | 0.02 |  |  |
| L-Trp | 0.01 |  |  |
| Chloride choline | 0.15 |  |  |
| Vitamin premix1 | 0.05 |  |  |
| Mineral premix2 | 0.30 |  |  |
| Total | 100.00 |  |  |

^1^ The vitamin premix provides the diet (per kg) with 9000 IU VA, 3000 IU VD_3_, 20 IU VE, 3 mg VK_3_, 1.5 mg VB_1_, 4 mg VB_2,_ 3 mg VB_6_, 0.02 mg VB_12_, 30 mg niacin, 15 mg pantothenic acid, 0.75 mg folic acid, and 0.1 mg biotin.

^2^ The mineral premix prodies the diet (per kg) with 100.0 mg Fe, 6.0 mg Cu, 100.0 mg Zn, 4.0 mg Mn, 0.14 mg I, 0.3 mg Se.

Supplementary Table 2 Sequences of primers and probes for qPCR

| **Gene** | **Primer sequence (5’ –3’)** |
| --- | --- |
| *β-Actin* | F: TGGAACGGTGAAGGTGACAGC |
|  | R: GCTTTTGGGAAGGCAGGGACT |
| *ZO-1* | F: TGGCATTATTCGCCTTCATAC |
|  | R:ATCGATATGGAGGAGGTGATATGG |
| *Claudin-1* | F: TCTTAGTTGCCACAGCATGG |
|  | R:CCAGTGAAGAGAGCCTGACC |
| *Occludin* | F: CTACTCGTCCAACGGGAAAG |
|  | R:ACGCCTCCAAGTTACCACTG |
| *FATP-1* | F:GGAGTAGAGGGCAAAGCAGG |
|  | R:AGGTCTGGCGTGGGTCAAAG |
| *SGLT-1* | F: GCAACAGCAAAGAGGAGCGTAT |
|  | R:GCCACAAAACAGGTCATAGGTC |
| *GLUT-2* | F: GACACGTTTTGGGTGTTCCG |
|  | R:GAGGCTAGCAGATGCCGTAG |
| *PETP1* | F: AAGATGAAATGTGAGCGTATGGG |
|  | R:TTACTCGGCTGGTTTTTTGAGTG |
| *PBD3* | F: CCTTCTCTTTGCCTTGCTCTT |
|  | R: GCCACTCACAGAACAGCTACC |
| *PBD114* | F: TGGTGGATCCTGAACGATGCT |
|  | R: AAACGCCCTCTGAATGCAGC |
| *Arpc2* | F: GTGCTCGGGACAATACCATCAA |
|  | R: CCCGCATACGTGTGTGAATATAGG |
| *ARPC3* | F: AGATGAAGTGATGAGGGCCTATTT |
|  | R: TCCACCACTTGCTGGGTTTAT |
| *WASL* | F: TCACCTTCCTCGGCAAGAAAT |
|  | R: CAAGACAAGCAACACCACTACAC |
| *ITGA5* | F: CAGACAACTTCACCCGGATTCT |
|  | R: TTGGTGAACTCGGCACTGAA |
| *ITGB1* | F: ACTCGGTGAATGGGAACAATGA |
|  | R: AGCAACCACACCAGCTACAA |
| *vcl* | F: CAAAGGCTGTGGCAGGAAAC |
|  | R: CAGGCTCCTGAGGTTGGAAG |

Supplementary Figure S1. Representative melting curves of qPCR amplification for all target genes analyzed in this study.
